# Human coronaviruses and therapeutic drug discovery

**DOI:** 10.1186/s40249-021-00812-9

**Published:** 2021-03-16

**Authors:** Lan-Gui Song, Qing-Xing Xie, Hui-Lin Lao, Zhi-Yue Lv

**Affiliations:** 1 The Eighth Affiliated Hospital, Sun Yat-Sen University, Shenzhen, Guangdong China; 2 Zhongshan School of Medicine, Sun Yat-Sen University, Guangzhou, Guangdong China; 3 NHC Key Laboratory of Control of Tropical Diseases, the First Affiliated Hospital, Hainan Medical University, Haikou, China; 4 Key Laboratory of Tropical Disease Control (Sun Yat-Sen University), Ministry of Education, Guangzhou, China

**Keywords:** Human coronavirus, Drug discovery, Drug development, SARS-CoV-2

## Abstract

**Background:**

Coronaviruses (CoVs) are distributed worldwide and have various susceptible hosts; CoVs infecting humans are called human coronaviruses (HCoVs). Although HCoV-specific drugs are still lacking, many potent targets for drug discovery are being explored, and many vigorously designed clinical trials are being carried out in an orderly manner. The aim of this review was to gain a comprehensive understanding of the current status of drug development against HCoVs, particularly severe acute respiratory syndrome coronavirus 2 (SARS-CoV-2).

**Main text:**

A scoping review was conducted by electronically searching research studies, reviews, and clinical trials in PubMed and the CNKI. Studies on HCoVs and therapeutic drug discovery published between January 2000 and October 2020 and in English or Chinese were included, and the information was summarized. Of the 3248 studies identified, 159 publication were finally included. Advances in drug development against HCoV, especially SARS-CoV-2, are summarized under three categories: antiviral drugs aimed at inhibiting the HCoV proliferation process, drugs acting on the host's immune system, and drugs derived from plants with potent activity. Furthermore, clinical trials of drugs targeting SARS-CoV-2 are summarized.

**Conclusions:**

During the spread of COVID-19 outbreak, great efforts have been made in therapeutic drug discovery against the virus, although the pharmacological effects and adverse reactions of some drugs under study are still unclear. However, well-designed high-quality studies are needed to further study the effectiveness and safety of these potential drugs so as to provide valid recommendations for better control of the COVID-19 pandemic.

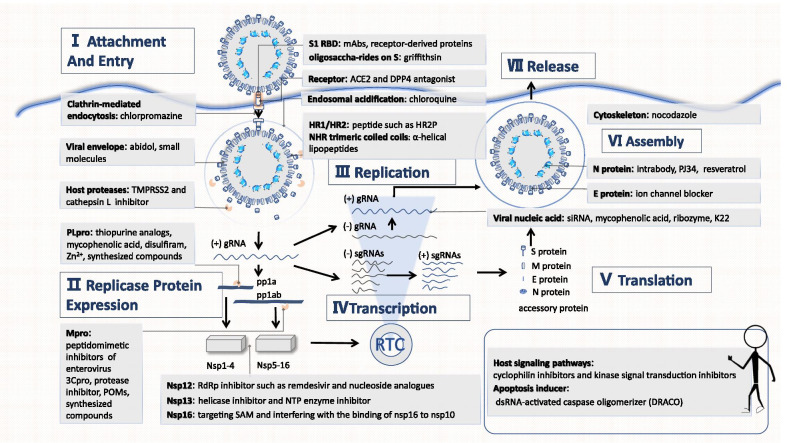

## Background

Coronaviruses (CoVs), which consist of nucleoproteins (N), envelope proteins (E), matrix proteins (M), spike proteins (S), and many non-structural proteins, are linear single-stranded RNA viruses [1]. CoVs are a large family of viruses with various susceptible hosts, including humans and many other animal species, such as camels, cattle, cats, and bats [2]; those infecting humans are called human coronaviruses (HCoVs). HCoVs include HCoV-229E, NL63, OC43, HKU1, severe acute respiratory syndrome coronavirus (SARS-CoV), Middle East respiratory syndrome coronavirus (MERS-CoV) and SARS-CoV-2 and are recognized to be important causes of respiratory tract infection [3, 4]. The former four types are considered common HCoVs and usually lead to mild to moderate upper respiratory tract illnesses [4], while the other three types are different. After the outbreaks of SARS in 2002 and MERS in 2012, the world experienced the coronavirus disease 2019 (COVID-19) pandemic caused by SARS-CoV-2 in 2020. Similar to SARS-CoV, SARS-CoV-2 appeared unexpectedly and spread throughout the world rapidly, with 56 623 643 confirmed cases and 1 355 963 deaths [5]. Fever and cough are the most common symptoms of COVID-19; patients infected with SARS-CoV-2 can develop dyspnoea a week after onset, and critical patients usually die from uncontrollable sepsis, respiratory failure, acute respiratory distress syndrome (ARDS) and septic shock [6]. Therapeutic interventions excluding virus-specific drugs, are often experiential or anecdotal and have not been tested in an integrated trial to provide sufficient and widely accepted evidence. The most common interventions include a combination of antivirals (such as ribavirin and lopinavir/ritonavir) and interferons (IFNs), corticosteroids, COVID-19 convalescent plasma and supportive treatment for critical patients [7].

Previous studies have revealed the invasion mechanism of HCoVs. In brief, S1 binds to the relevant receptor and induces endocytosis, then the conformation of the S2 subunit changes. The viral envelope fuses with the endosomal membrane and releases the nucleocapsid or viral genome [8]. Genomic RNA (gRNA) serves as a translation template for polyproteins pp1a and pp1ab, which are automatically hydrolysed into various non-structural proteins (NSPs), such as papain-like protease (PLpro), 3C-like protease (Mpro), and RNA-dependent RNA polymerase (RdRp). Full-length gRNA is replicated by negative sense intermediates and transcribed into subgenomic RNA (sgRNA). sgRNA encodes the structural proteins of the virus (N, M, E, and S) as well as helper proteins (e.g., 3, 4a, 4B, 5, and 8b). Particle assembly occurs in the ER-Golgi intermediate compartment (ERGIC) and is then released in the vesicle via the secretory pathway [3]. Interruption of the proliferation process might help cure patients and disrupt transmission. As the understanding of both the biological characteristics and pathogenicity of HCoVs has deepened, many potent targets for drug discovery have been explored, such as inhibiting HCoV invasion and strengthening host immune defences. In addition, traditional Chinese medicine might be effective in the fight against HCoVs. To establish additional evidence supporting recommended treatment strategies, some drugs, such as remdesivir, favipiravir, lopinavir/ritonavir, arbidol/umifenovir, and hydroxychloroquine, have been tested in vigorously designed clinical trials. Herein, we review the progress in therapeutic drug discovery and development, including drugs that inhibit the CoV proliferation process (attachment and entry, replicase expression, replication, transcription and translation, assembly and release), antiviral drugs that affect the action of the host's immune system, and drugs derived from plants with potent activity, in order to accelerate drug discovery and development, especially during the current pandemic.

## Main text

### Methodology

#### Search strategy

We searched two databases: PubMed (https://www.ncbi.nlm.nih.gov/pubmed/) and CNKI (www.cnki.net). We searched for coronavirus (or HCoV) and important components (such as S protein, PLpro, Mpro, NSPs, RNA, N protein, E protein, host factors) and drugs (or Chinese medicine, plant derivates, or research or treatment). References of studies retrieved were cross checked as well. All the search results were evaluated. First, the titles and abstracts were screened to identify relevant studies; then, full texts were evaluated carefully to determine eligibility for inclusion. The complete search and selection processes were performed by two independent researchers. Any disagreements were resolved through consultation with a third researcher or team discussion until consensus was reached.

#### Inclusion criteria

(1) The target coronaviruses were HCoVs, with special attention to highly pathogenic HCoVs; (2) The studied drugs included newly developed targeted drugs, broad-spectrum antiviral drugs, small-molecule compounds, plant derivatives, etc.; (3) the research performed included in vivo or in vitro tests, clinical trials, or literature reviews; (4) the publication language was English or Chinese; (5) the literature type was an article, review, or clinical trial; and (6) the publication time was from January 1, 2000, to October 27, 2020.

#### Exclusion criteria

(1) Duplicate studies; (2) studies for which the full text was unavailable; (3) news, reports, interviews, comments, patents, letters, or case reports; and (4) reviews or studies with the aim of elucidating the impact of coronavirus infection on the underlying diseases and their treatment in a target population.

#### Data extraction, summary, and analysis

We classified the selected documents according to the following categories: (1) antiviral drugs intended to inhibit the HCoV proliferation process; (2) antiviral drugs that affect the action of the host's immune system; and (3) antiviral drugs derived from plants with potent activity. All articles were processed using NoteExpress V 3.0 (Beijing Aegean Technology Co., Ltd., Beijing, China).

## Results

### The scoping process

**Fig. 1:**
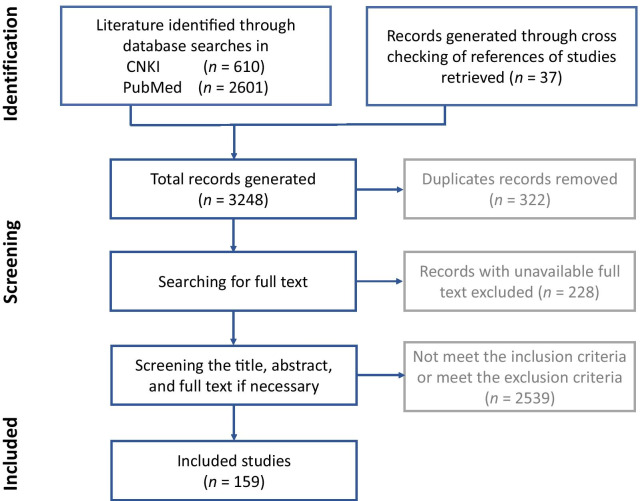
PRISMA flow diagram of the scoping review process

A total of 3248 records were retrieved. After excluding 322 duplicate records, 228 records with unavailable full texts, and 2539 records that met the exclusion criteria mentioned above, 159 records were finally included in this review. A flow diagram of the study selection process is shown in Fig. 1.

**Fig. 2:**
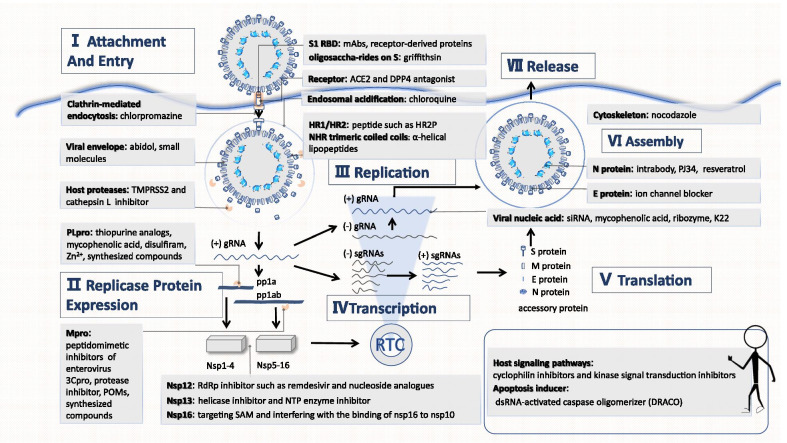
The CoV proliferation process. TMPRSS2: Transmembrane protease serine 2; Zn^2+^: Zinc ion; PLpro: Papain-like protease; Mpro: 3C-like protease; 3Cpro: 3C protease; POMs: Polyoxometalates; Nsps: non-structural proteins; NTP: Nucleoside triphosphate; SAM: S-adenosyl-l-methionine; RBD: Receptor binding domain; mAb: monoclonal antibodies; ACE2: Angiotensin converting enzyme 2; DPP4: dipeptidyl peptidase 4; HR1: Heptad repeat 1 domain; HR2: Heptad repeat 2 domain; gRNA: genomic RNA; sgRNA: subgenomic RNA; siRNA: small interfering RNA; NHR: N-terminal heptad repeat; dsRNA: Double-stranded RNA

The proliferation process as well as the key targets of CoVs are presented (Fig. 2). A summary of the progress in therapeutic drug discovery and development targeting HCoVs is demonstrated below and is divided into three sections: drugs that inhibit HCoV proliferation (attachment and entry, replicase expression, replication, transcription and translation, assembly and release), antiviral drugs that affect the actions of the host's immune system, and drugs derived from plants with potent activity.

### Blocking the HCoV proliferation process is the key to identify effective drugs against the virus

The drugs that target the CoV proliferation process are summarized in Table 1.

**Table 1:** Drugs aiming at the proliferation process

Step	Target		Drugs		Type	Reference number
			Category	Example		
Attachment and entry	Block receptor binding	S1-RBD	mAbs	m336, m337 and m338	MERS-CoV	[9]
				Human-derived SARS mAbs	SARS-CoV	[10, 11]
			Receptor-derived proteins	P4 and P5 peptides	SARS-CoV	[12]
		Receptor	ACE2 antagonist	NAAE	SARS-CoV	[13]
			DPP4 antagonist	Adenosine deaminase, anti-DPP4 mAbs	MERS-CoV	[14, 15]
		Oligosaccharide-rides on S	Antiviral protein	Griffithsin	SARS-CoV, MERS-CoV, HCoV‑229E, HCoV‑OC43	[16]
	Block endocytosis	Host proteases that cleavage S protein	TMPRSS2 inhibitor	Camostat, nafamostat	SARS-CoV,; MERS-CoV	[17, 18]
			Cathepsin L inhibitor	Teicoplanin, dalbavancin	SARS-CoV, MERS-CoV, SARS-CoV-2	[19]
		HR1/HR2	Peptide	HR2P and P1 peptide	MERS-CoV	[20, 21]
				229E-HR1P and 229E-HR2P	HCoV-229E	[22]
				OC43-HR2P	Broad spectrum	[23]
		Viral envelope	Antiviral drug	arbidol	Broad spectrum	[24]
			Small molecules	LJ001		[25]
		NHR trimeric coiled coils	α-helical lipopeptides		MERS-CoV	[26]
		Endosomal acidification	Antimalarial	Chloroquine, chloroquine phosphate	SARS-CoV-2	[30–32]
		Clathrin-mediated endocytosis	Antagonist of DA receptor	Chlorpromazine	MERS-CoV	[30]
Replicase protein expression	PLpro; (Papain-like protease); (nsp3)		Thiopurine analogs	6-mercaptopurine (6MP) and 6-thioguanine (6TG)	MERS-CoV	[33]
			Immunosuppressive drug	Mycophenolic acid	MERS-CoV	[33]
			Alcohol-aversive drug	Disulfiram	SARS-CoV, MERS-CoV	[34]
			Protease inhibitor	Zinc ion (Zn^2+^) and zinc conjugate inhibitors	SARS-CoV	[35]
			Compound	F2124-0890	SARS-CoV, MERS-CoV	[36]
	Mpro; (3C-like protease); (nsp5)		Peptidomimetic inhibitors of enterovirus 3Cpro	6b, 6c and 6d	SARS-CoV, MERS-CoV	[37]
			1,2,3-triazole derivatives	Compounds 14d, 14n, 14q, 18f and 18i	HCoV-229E	[38]
			Protease inhibitor	Lopinavir/ritonavir	SARS-CoV, MERS-CoV, HCoV-229E	[30, 39, 46, 48, 49]
				Darunavir/cobicistat	SARS-CoV-2	NCT04252274
			Protease inhibitor	Ti-containing polyoxometalates (POMs)	SARS-CoV	[40]
			Derivates of pyrithiobac (PTB)	Compound 6–5	SARS-CoV	[41]
			Peptide; mimic inhibitor	N3	Broad spectrum	[42]
			Synthesized compounds	Derivates of isatin, piperazineare and phenylisoserine	SARS-CoV	[44, 45]
				Derivates of piperidine	MERS-CoV	[43]
Replication, transcription and translation	NSPs	NSP12	RdRp inhibitor	Remdesivir	Broad spectrum	[50–54]
				Doubly flexible nucleoside analogues such as compound 2	Broad spectrum	[55]
				Galidesivir (synthetic adenosine analogue)	Broad spectrum	[56]
				6′-fluorinated-aristeromycin analogues	Broad spectrum	[57]
				Favipiravir	broad spectrum	[58, 59]
		NSP13	Helicase inhibitor	Aryl diketoacids (ADK), SSYA10-001, halogenated triazole compounds	SARS-CoV, MERS-CoV	[61, 62, 65]
			NTP enzyme inhibitor	Bananins, 2,6-bis-arylmethyloxy-5-hydroxychromones	SARS-CoV, SARS-associated CoV	[63, 64]
		NSP16	Drug targeting SAM	S-adenosine-1 homocysteine	SARS-CoV, HCoV 229E	[66]
				Paclitaxel		[66]
				Aurintricarboxylic acid (ATA)		[66]
			Drug interfering with the binding of NSP16 to NSP10	The peptide chain reversely designed according to the sequence of nsp16′s binding domain		[66]
	Host signaling pathways		Cyclophilin inhibitors	Cyclosporine, alisporivir	SARS-CoV, MERS- CoV, HCoV-NL63	[67–69]
			Kinase signal transduction inhibitors	Trametinib, imatinib	MERS-CoV	[3, 70]
	Viral nucleic acid or RNA synthesis complex		siRNA	Specific siRNAs targeting the S/M/E/N/accessory protein gene	SARS-CoV, MERS-CoV	[71–76]
			Immunosuppressive drug	Mycophenolic acid	MERS-CoV	[77]
			Ribozyme	Synthetic chimeric DNA–RNA hammerhead ribozyme	SARS-CoV	[78]
			Synthesis	K22 (targeting membrane-bound viral RNA)	Broad spectrum	[79]
			Apoptosis inducer	dsRNA-activated caspase oligomerizer (DRACO)	Broad spectrum	[80]
	N protein		Compound	*N*-(6-oxo-5,6-dihydrophenanthridin-2-yl) (*N*,*N*-dimethylamino)acetamide hydrochloride (PJ34)	HCoV-OC43	[81]
			Intrabody	Fibronectin-based intrabodies	SARS-CoV	[82]
			Inhibitor	Resveratrol	MERS-CoV	[83]
Assembly and release	E protein		Ion channel blocker	Hexamethylene amiloride	HCoV-229E	[84]
	Cytoskeleton		Filament depolymerizing drug	NOC (nocodazole)	HCoV-229E, HCoV-NL6	[3, 85]

RBD: receptor binding domain; mAbs: monoclonal antibodies; SARS: Severe acute respiratory syndrome; MERS: Middle East respiratory syndrome; ACE2: Angiotensin converting enzyme 2; DPP4: dipeptidyl peptidase 4; NHR: N-terminal heptad repeat; HR1: Heptad repeat 1 domain; HR2: Heptad repeat 2 domain; DA: dopamine; 6MP: 6-mercaptopurine; 6-TG: 6-thioguanine; Zn^2+^: zinc ion; POM: polyoxometalate; PTB: derivates of pyrithiobac; NAAE: *N*-(2-aminoethyl)-1-aziridineethanamine; TMPRSS2: transmembrane protease serine 2; Nsps: non-structural proteins; RdRp: RNA-dependent RNA polymerase; ADK: aryl diketoacids; NTP: nucleoside triphosphate; SAM: S-adenosyl-l-methionine; ATA: aurintricarboxylic acid; dsRNA: double-stranded RNA; DRACO: dsRNA-activated caspase oligomerizer; siRNA: small interfering RNAs; PJ34: *N*-(6-oxo-5,6-dihydrophenanthridin-2-yl) (*N*,*N*-dimethylamino) acetamide hydrochloride; NOC: nocodazole

#### Inhibition of attachment and entry

The process of invasion can be divided into receptor binding and endocytosis [2]. Drugs developed from viruses or host structures that participate in the above two processes have the potential to block virus invasion.

##### Prevention of receptor binding

The receptor binding domain (RBD) is the domain that binds to the receptor during HCoV invasion; RBDs have substantial diversity [2]. There have been many studies on therapeutic monoclonal antibodies (mAbs), including m336, m337 and m338, that target the RBD to prevent MERS-CoV invasion [9] and human-derived SARS-CoV [10, 11]. In addition to mAbs, receptor-derived proteins based on the ligand-binding domain, such as P4 and P5 peptides, can be utilized to competitively bind to the RBD [12].

To enter a cell, the RBD needs to bind to a receptor. Thus, in theory, drugs that compete with RBDs for receptor binding sites, such as *N*-(2-aminoethyl)-1-aziridineethanamine (NAAE) [13] and anti-dipeptidyl peptidase 4 (DPP4) mAbs, can block CoV invasion [14, 15]. However, considering that the receptors on the host cell surface also play an important role in the normal metabolism and function of the cell, the development of such drugs should take into account their impacts on the body, such as hypotensive and hypoglycaemic effects. Griffithsin (GRFT) can specifically bind to the glycosyl groups of protein S, thereby inhibiting virus invasion [16].

##### Prevention of endocytosis

Inhibitors of host proteases that cleave protein S, such as transmembrane protease serine 2 (TMPRSS2) inhibitors (camostat [17], nafamostat [18]) and cathepsin L inhibitors (teicoplanin, dalbavancin), can prevent exposure and insertion of the hydrophobic end of S2 into the endosomal membrane [19]. Drugs targeting HR1/HR2, such as HR2P, P1 peptide, 229E-HR1P, 229E-HR2P and OC43-HR2P peptide [20–23], can prevent the formation of the 6-helix bundle structure, thereby inhibiting the fusion of the viral envelope with the endosomal membrane. Furthermore, arbidol [24], LJ001 [25], and NHR trimeric coiled coil alpha-helical lipopeptides prevent enveloped virus-cell membrane fusion [26]. Arbidol was tested in a clinical trial and appeared to reduce the SARS-CoV-2 RNA load [27]; however, other studies revealed that Arbidol did not improve the clinical outcomes of patients or SARS-CoV-2 elimination [28, 29]. Attention should be paid to host factors that affect endocytosis. For instance, chloroquine inhibits endosomal acidification [30, 31], and chlorpromazine inhibits clathrin-mediated endocytosis [30]. Recently, chloroquine phosphate has been recommended by Chinese scholars for the treatment of SARS-CoV-2 [32], but some studies have shown that hydroxychloroquine induces cardiotoxicity [33, 34].

#### Inhibition of replicase expression

In the replication cycle, CoV RNA is first translated into two polyproteins, pp1a and pp1ab, which are then hydrolysed to generate sixteen NSPs with various functions [2]. Certain NSPs are essential for virus replication and transcription.

Papain-like protease (namely, NSP3 and PLpro) and Achilles’ heel 3C-like protease (also known as NSP5 and Mpro) play a vital role in hydrolysing polyproteins to generate NSPs [2]. Hence, inhibitors of the two proteases can block the generation of NSPs.

##### PLpro

Drugs targeting PLpro include the thiopurine analogues 6-mercaptopurine (6MP) and 6-thioguanine (6TG), mycophenolic acid [35], disulfiram [36], zinc ion (Zn^2+^) and zinc conjugate inhibitors [37], as well as F2124-0890 [38].

##### Mpro

Drugs targeting Mpro include peptidomimetic inhibitors of enterovirus 3Cpro (6b, 6c and 6d) [39], a novel series of fused 1,2,3-triazoles [40], lopinavir/ritonavir, [30, 41], Ti-containing polyoxometalates (POMs) [42], compounds 6–5 derived from pyrithiobac (PTB) [43], some molecules such as N3 [44], and synthesized compounds (derived from isatin, piperazine, piperidine and phenylisoserine) [45–47]. According to a randomized control trial published in *The Lancet*, lopinavir–ritonavir was not associated with survival improvement or mortality reduction [48, 49], so the World Health Organization (WHO) terminated related experiments.

#### Inhibition of replication, transcription and translation

CoV replicase synthesizes the full-length antisense genome using gRNA as a template and then synthesizes new gRNA according to the sequence of the antisense RNA. Thereafter, with the help of RNA polymerase and certain transcription factors, the virus recognizes specific transcriptional regulatory sequences (TRSs) with "discontinuous transcription" and selectively transcribes all components that make up a mature mRNA. Finally, mRNA is translated into a variety of structural proteins (nucleocapsid protein N, membrane protein M, envelope protein E, and spike protein S) and accessory proteins (such as 3, 4a, 4b, 5 and 8b) [2].

##### Essential NSPs

A variety of NSPs play significant roles in the replication process, whereas drugs targeting them are limited.

NSP12 (RdRp inhibitors): The NSP12 (RdRp) inhibitor remdesivir prevents viral replication and thus reduces the viral load in patients [50]. However, two recent clinical trials have reached two different conclusions. A study published in *the Lancet* revealed that the drug is not effective [51–53], while a study published in *New England Journal of Medicine* showed that the drug shortened the length of hospitalization and virus removal time. However, this paper was withdrawn for many reasons [54]. Other drugs include a series of doubly flexible nucleoside analogues [55], galidesivir (BCX4430) [56], a novel synthetic adenosine analogue, 6′-fluorinated-aristeromycin analogues [57], and favipiravir. Favipiravir has been associated with improvement in chest CT findings [58, 59]. However, the broad-spectrum antiviral drug ribavirin had no significant effects on clinical outcomes when administered alone for the treatment of SARS [60].

NSP13: With the activity of both nucleotide helicase and nucleoside triphosphate (NTP) enzymes, NSP13 functions to unravel the dsRNA helix. Drugs targeting NSP13 not only alter helicase activity [such as aryl diketoacids (ADK) and SSYA10-001] [61, 62], but also affect NTP enzyme activity [such as bananins and 2,6-bis-arylmethyloxy-5-hydroxychromones] [63, 64]. Furthermore, molecular docking results showed that 16 halogenated triazole compounds could bind to NSP13, with inhibitory effects [65].

NSP16 [S-adenosyl-l-methionine (SAM)-dependent 2′O-MTase]: Drug action mechanisms can be divided into two types: direct termination of 2′O-MTase activity through the alteration of SAM (drugs that utilize this mechanism include S-adenosine-1 homocysteine, paclitaxel, and aurintricarboxylic acid (ATA) [66]) or alteration of 2′O-MTase activity by interfering with the binding of NSP16 to NSP10 (drugs that utilize this mechanism include complementary reverse peptides designed according to the sequence of the NSP16 binding domain [66]).

##### Host signalling pathways

Certain host signalling pathways are essential for viral replication [3]. The cyclophilin inhibitors cyclosporine and alisporivir regulate the interactions of cyclophilin with NSP1 and the calcineurin-NFAT pathway [67–69]. Kinase signal transduction inhibitors, such as trametinib and imatinib, block the ABL1, ERK–MAPK and PI3K–AKT–mTOR pathways, potentially preventing early virus invasion and resulting immune disorders [3, 70].

##### Viral nucleic acids and RNA synthesis complex

Various small interfering RNAs (siRNAs) can interfere with viral replication as well as the expression of structural proteins and accessory proteins [71–76]. Mycophenolic acid may inhibit viral nucleic acid synthesis [77], but it is advisable to combine it with an interferon since its immunosuppressive effect may create an environment amenable to virus replication and dissemination. In addition, a synthetic chimaeric DNA–RNA hammerhead ribozyme can suppress the expression of SARS-CoV RNA [78]. Moreover, K22 can suppress RNA synthesis by inhibiting the formation of double membrane vesicles (DMVs) [79]. Finally, given the existence of replication intermediates, dsRNA-activated caspase oligomerizer (DRACO) can selectively induce apoptosis in cells containing viral dsRNA [80].

##### Protein N

Newly generated RNA needs to bind to protein N to form a nucleocapsid for stability; protein N also plays an important role in the normal replication and transcription of gRNA [2]. Therefore, drugs targeting protein N, such as fibronectin-based intrabodies and the inhibitors PJ34 and resveratrol, may influence these processes [81–83].

#### Inhibition of assembly and release

Viral assembly occurs in the ERGIC, where proteins M and E play important roles [2]. Hexamethylene amiloride [84] blocks the E protein ion channel. CoV particles in ERGIC are transported through the secretory pathway in vesicles and released through exocytosis [2].

The interactions between the cytoskeleton and structural proteins are essential for the assembly and release of CoVs [3]. For example, nocodazole may reduce the amount of transmissible gastroenteritis virus (TGEV), which belongs to the genus α-CoV and shares a similar assembly and release mechanisms with HCoV-229E and HCoV-NL63, particles released from the body [85]. Nonetheless, the advantages and disadvantages must be considered before administering the drug due to the significant role of the cytoskeleton in the normal metabolism and functioning of cells.

### Drugs that affect the action of the host's immune system could help relieve the symptoms

#### Innate immunity

Complement activation and IFNs are believed to play an active role in the innate immune response against HCoVs.

##### Complement activation

Inhibition of complement activation alleviates acute lung injury induced by SARS-CoV and MERS-CoV infection. For instance, anti-C5aR antibody treatment resulted in decreased viral replication in lung tissues in hDPP4-transgenic mice infected with MERS-CoV. SARS-CoV-infected C3^−/−^ mice exhibited significantly less weight loss and less respiratory dysfunction despite an equivalent viral load in the lungs [86–88].

IFNs. IFN-α/β (IFN-1) is an important component of innate immune defence, which protects mammalian hosts from viral infection [89]. While mild HCoV infections, such as infection by HCoV-229E, typically induce a high level of IFN-I production [90], SARS-CoV and MERS-CoV were shown to suppress the activation of the host innate immune response by inhibiting interferon production or signalling. Several structural proteins (M and N) [91–93], NSPs (NSP1 and NSP3) [94–96], and accessory proteins of SARS-CoV and/or MERS-CoV were identified as IFN antagonists [92]. In addition to inhibiting CoV replication, drugs targeting these proteins may work by unblocking IFN suppression by the CoV. IFN has been clinically indicated to be effective for the treatment of SARS-CoV and MERS-CoV. In clinical treatment, the routine use of IFNs is not recommended for SARS-CoV treatment [97]. IFNs are usually administered in combination with other drugs, such as IFN-β-1b combined with lopinavir/ritonavir [98] or ribavirin and IFN-α combined with lopinavir/ritonavir [99], for MERS-CoV treatment. In severe to critical COVID-19 patients, early treatment with IFN-α2b can reduce in-hospital mortality, but it has no significant benefit in moderately ill patients [100].

#### Cell-mediated immunity

Lymphocytopenia is commonly observed in patients infected with SARS-CoV [97], MERS-CoV [101], or SARS-CoV-2 [102], but the mechanism remains unclear. Human T cells are highly susceptible to MERS-CoV infection. Studies have demonstrated that MERS-CoV persists in T cell-deficient mice but is cleared in B cell-deficient mice, suggesting that T cells play a critical role in MERS-CoV clearance [103]. SARS-CoV-specific T cells also play important roles in the recognition and clearance of infected cells [104].

#### Humoural immunity

Antibodies play an important role in preventing CoV infection. Antibody production against protein S was less in SARS-CoV-infected patients with fatal outcomes than in non-severe patients [105]. The level and presence of antibodies are related to the clinical severity of SARS and MERS [106, 107]. Experiments have shown that antibody therapy improves symptoms and promotes recovery. SARS-CoV-specific monoclonal antibodies include human mAb CR3014 [10], CR3022 [108], and 5H10 [109]. MERS-CoV-specific monoclonal antibodies include m336 [110], REGN3051, REGN3048 [111], 3B11-N [112], LCA60 [113], MCA1 [114], MERS-4, MERS-27 [115], MERS-GD27, and MERS-GD33 [116]. Serum cross-reaction is important for both detection and treatment. Studies have shown the absence of cross-reactivity between SARS-CoV and MERS-CoV. The SARS-CoV-specific human monoclonal antibody CR3022 can effectively bind to the RBD of SARS-CoV-2 [117].

#### Convalescent plasma

Convalescent plasma therapy may be beneficial for patients with early SARS infection because it provides antibodies from convalescent patients [118], but evidence of its efficacy in MERS-CoV patients is still lacking. It is recommended for the treatment of rapidly progressing, severe and critical cases of SARS-CoV-2 infection [99], but it is limited by safety concerns and inadequate sources. Trials indicate that convalescent plasma is most effective in reducing mortality when administered in the early stage of infection, but it does not significantly shorten the time to recovery [119, 120].

#### Glucocorticoids

Corticosteroids not only suppress lung inflammation but also inhibit immune responses and pathogen clearance. Available observational data suggest impaired clearance of SARS-CoV and MERS-CoV as well as increased complication rates in survivors receiving corticosteroid therapy. Therefore, it is not advisable to administer corticosteroid treatment in patients with SARS-CoV-2-associated lung injury or shock outside of a clinical trial setting [121]. Recent clinical trials suggest that early, low-dose methylprednisolone administered in the short term improved clinical outcomes and reduced mortality in severe COVID-19 patients [122–124]. Guidelines from China recommend that glucocorticoids should be used in the short term as appropriate in patients with progressive deterioration of the oxygenation index, rapid radiographic development, and excessive activation of the inflammatory response [99].

#### IL-6 receptor inhibitors

IL-6 plays an important role in the development of a cytokine storm. As an IL-6 receptor inhibitor, tocilizumab does not prevent the disease from progressing, but it can reduce the symptoms of serious infection [125–127].

Clinical trials of drugs targeting SARS-CoV-2 are summarized in Table 2.

**Table 2:** Published clinical trials of drugs against SARS-CoV-2

Step	Target	Drug	Result		Population	Methodology	ID	Reference number
			Positive	Negative				
Attachment and entry	Viral envelope	Arbidol/umifenovir	N/A	Umifenovir did not improve the prognosis or accelerate SARS-CoV-2 clearance in non-ICU patients	81 moderates to severe COVID-19 patients (umifenovir vs control = 45: 36)	Single-centre, retrospective	N/A	[29]
			The ability of arbidol to reduce SARS-CoV-2 RNA load is better than lopinavir–ritonavir	N/A	50 COVID-19 patients (lopinavir/ritonavir vs arbidol = 34:16)	N/A	N/A	[27]
	Endosomal acidification	Hydroxychloroquine	N/A	Hydroxychloroquine did not induce SARS-CoV-2 negative conversion. It has cardiotoxicity	150 patients with mainly persistent mild to moderate COVID-19 (hydroxychloroquine + standard of care vs standard of care alone = 75:75)	Multicenter, open label, randomized controlled	ChiCTR2000029868	[33]
		Chloroquine diphosphate	N/A	Chloroquine diphosphate did not reduce mortality and may extend QT intervals	81 patients (high-dosage vs low-dosage group = 41:40)	Parallel, double-masked, randomized, phase IIb	N/A	[34]
Replicase protein expression	Mpro (3C-like protease) (nsp5)	Lopinavir/ritonavir	N/A	Lopinavir–ritonavir did not reduce mortality or the time to clinical improvement	199 COVID-19 adults (lopinavir–ritonavir:standard-care = 99:100)	Randomized, controlled, open-label	ChiCTR2000029308	[49]
			N/A	Lopinavir–ritonavir did not reduce mortality or prevent progression	5040 (lopinavir/ritonavir:usual care = 1616:3424)	Randomised, controlled, open-label, platform	NCT04381936	[48]
Replication, transcription and translation	Nsp12	Remdesivir	By day 28, 10-day remdesivir group had a better clinical status distribution than standard care group	Remdesivir did not reduce the length of hospitalization or oxygen therapy	584 moderate COVID-19 patients (10-day remdesivir:5-day remdesivir:standard care = 197:199:200)	Randomized, controlled, open-label	NCT04292730	[53]
			Remdesivir reduces the duration of hospitalization and infection	N/A	1059 COVID-19 adults with lower respiratory tract infection (remdesivir:placebo = 538:521)	Double-blind, randomized, placebo-controlled	NCT04280705	[54]
			N/A	There is no significant clinical difference between a 5-day course and a 10-day course of remdesivir	397 severe COVID-19 patients (5-day remdesivir:10-day remdesivir = 200:197)	Randomized, open-label	NCT04292899	[52]
			N/A	Remdesivir did not reduce time to clinical improvement	237 COVID-19 adults (10- day remdesivir vs placebo = 158:79)	Randomised, double-blind, placebo-controlled, multicentre	NCT04257656	[51]
		Favipiravir	Favipiravir leads to faster viral clearance and better chest CT changes than patients treated with lopinavir/ritonavir	N/A	80 COVID-19 patients (favipiravir vs lopinavir/ritonavir = 35:45)	Open-label comparative controlled	ChiCTR2000029600	[59]
Immunology	IFN supplement	IFN-α2b	Among severe to critical COVID-19 patients, early treatment with IFN-α2b reduced in-hospital mortality	IFN-α2b did not benefit significantly in moderately ill patients	242 (IFN + LPV/r, IFN + UFV, IFN alone) of 446 COVID-19 patients received IFN-α2b	Retrospective, multicenter		[100]
	Protective antibody supplement	Convalescent plasma	Convalescent plasma is most effective in early application and reduces mortality	N/A	39 patients with severe to life-threatening COVID-19 (convalescent plasma vs controls = 1:4 and 1:2 ratios)	Retrospective, propensity score–matched case–control	N/A	[119]
			Convalescent plasma was associated with antiviral activity	Convalescent plasma did not significantly reduce time to the clinical improvement	103 patients with severe to life-threatening COVID-19 (Convalescent plasma in addition to standard treatment vs standard treatment alone = 52:51)	Open-label, multicenter, randomized, prospective	ChiCTR2000029757	[120]
	Cytokine storm	Corticosteroids	Early, low-dose and short-term application of methylprednisolone helped reach better clinical outcomes in severe patients with COVID-19 pneumonia	N/A	46 severe patients with COVID-19 pneumonia (26 of them received extra low-dose and short-term methylprednisolone treatment)	Retrospective	N/A	[122]
			For severe COVID-19 patients, methylprednisolone pulse promoted clinical improvements and reduced mortality	N/A	68 severe COVID-19 patients (methylprednisolone vs standard care alone = 34:34)	Single-blind, randomized, controlled	N/A	[123]
	IL-6R	Tocilizumab	N/A	Tocilizumab showed no benefit on disease progression compared with standard care. (early shutdown)	126 adults with COVID-19 pneumonia and PaO_2_/FIO_2_ ratio between 200 and 300 mmHg (tocilizumab vs supportive care = 60:66)	Prospective, open-label, randomized	NCT04346355	[127]
			N/A	Tocilizumab did not promote clinical improvements or reduced mortality	131 COVID-19 patients with moderate or severe pneumonia requiring oxygen but without ventilation or admission to the ICU (tocilizumab vs usual care alone = 64:67)	Multicenter, open-label, Bayesian randomized	NCT04331808	[126]
			Tocilizumab reduced serious infections	Tocilizumab did not prevent intubation or death in moderately ill hospitalized patients with COVID-19	243 hospitalized COVID-19 patients (standard care + tocilizumabvs vs standard care + placebo = 162:81)	Randomized, double-blind, placebo-controlled	NCT04356937	[125]

N/A: not applicable; ICU: intensive care unit; SARS-CoV-2: Severe acute respiratory syndrome coronavirus 2; COVID-19: Coronavirus disease 2019; CT: computed tomography; IFN: Interferon; IL: interleukin; PaO_2_/FIO_2_: OXYGENATION index

### Plant-derived Chinese medicine might have antiviral effect

#### Single Chinese medicines and their associated active ingredients

##### SARS-CoV-2

The traditional Chinese medicine components that might block the binding regions of grid3 and grid4 between angiotensin converting enzyme 2 (ACE2) and viral protein S include *Folium mori, Atractylodes lancea, Fritillaria, Zingiber officinale, Lonicerae japonicae* flo*s, Forsythia suspensa,* and *Amomum tsao-ko* [143]. SARS-CoV-2 leads to the downregulation of ACE2 upon binding to the receptor, thus disrupting normal regulation of the ACE-Ang II and ACE2-Ang-(1–7) axes, consequently inducing multiple organ damage. *Astragalus, Panax ginseng, Dioscorea* spp*.,* and arecae semen, which are major components of traditional Chinese medicine preparations for COVID-19 pneumonia, have shown a regulatory effect on the renin–angiotensin–aldosterone system (RAAS) [144]. *Quercetin* and its derivatives have strong binding ability to ACE2 and IL-6R and have the potential to inhibit the cytokine storm by blocking SARS-CoV-2 and IL-6 binding. In addition, licorice*,* ephedra*, Bupleurum* root, etc., also have different IL-6R binding abilities [145–147]. Saikoside A and saikoside D had good affinity with Mpro and ACE2 of SARS-CoV-2 [148]. The binding strengths of baicalein and SARS-CoV-2 Mpro are the same as those of lopinavir and remdesivir, and the bond to ACE2 is relatively stable [149]. Liquiritin apioside, iridin, liquiritin, forsythiaside, procyanidin B-5,3′-*o*-gallate and saikosaponin C are latent active RdRp inhibitors, and their flavonoid structures may be potential active groups that induce RdRp inhibition [150]. Aster pentapeptide A, ligustrazine, salvianolic acid B*,* etc., have potential inhibitory effects on SARS-COV-2 Mpro, while gingerol, ginnol, ferulic acid, etc., have potential inhibitory effects on SARS-COV-2 PLpro [151]. Hypericin and baicalein can bind to SARS-CoV-2 NSP14 and interact with key amino acid residues in the active centre [152].

##### SARS-CoV

Glycyrrhizin [128, 129] is capable of inhibiting the invasion and replication of SARS-CoV in vitro, and various derivatives [130] (such as the introduction of 2-acetamide-glucan amine into the glycyrrhizin chain) may account for increased anti-SARS-CoV activity along with enhanced cytotoxicity. Lycorine from *Lycoris radiata* and ZZ-1 [131, 132] may inhibit SARS-CoV replication. Polysaccharides and ethyl acetate extracts from *Houttuynia cordata* act on the body's immune system with anti-complement activity, among which afzerin and quercetin also have antipyretic effects [133]. *Houttuynia cordata* also promotes the inhibition of RdRp [134]. Lung injury caused by SARS-CoV is associated with inflammation due to cytokine storms and neutrophil infiltration. Thus, inhibiting cAMP-PDE, which plays a key role in the inflammatory response, may help prevent inflammation. *Rhizoma phragmitis*, *Folium isatidis*, honeysuckle, forsythia, perilla leaf, mint and *Astragalus* significantly inhibit cAMP-PDE activity [135]. *Multiflorum* and *Rheum rhabarbarum*, specifically its extract-derived component emodin, affect virus invasion [136]. Protease inhibitors of natural origin include 3CLpro inhibitors (such as quinone-methide triterpenes extracted from *Tripterygium regelii* [137], dieckol from *Ecklonia cava* [138], and extracts of *Houttuynia cordata* and *Rheum rhabarbarum* [134, 136]) and PLpro inhibitors (such as diarylheptanoids from *Alnus japonica* [139], and phenolic phytochemicals from the seeds of *Psoralea corylifolia* [140]). Finally, chalcone 6 from *Angelica keiskei* and tanshinones from *Salvia miltiorrhiza* are capable of inhibiting both 3CLpro and PLpro [141, 142].

##### MERS-CoV

Silvestrol [153], an inhibitor of eIF4A, can inhibit viral mRNA cap-dependent translation. In addition, research on MERS-CoV 3CLpro suggests that flavonoids such as herbacetin, isobavachalcone, quercetin 3-β-D-glucoside and helichrysetin [154] can act as inhibitors.

##### HCoV-229E

3β-Friedelanol [155], a triterpenoid extracted from the leaves of *Euphorbia neriifolia*, showed stronger antiviral activity than actinomycin D, the positive control. Furthermore, silvestrol [153], an eIF4A inhibitor, affects the translation of HCoV-229E.

##### HCoV-NL63

Caffeic acid, which is related to the ethanol extract of *Sambucus FormosanaNakai* [156], has been confirmed to have a significant inhibitory effect on the invasion of HCoV-NL63, possibly by directly interfering with the binding of HCoV-NL63 to ACE2 and co-receptors, such as heparin sulphate proteoglycan.

#### Compound traditional Chinese medicines

##### SARS-CoV-2

Based on the Chinese COVID-19 diagnosis and treatment scheme, ageratum upright capsules (in the form of pills, water, or oral liquid), Jinhuaqinggan particles, Lianhuaqingwen capsules (particles) and Shufengjiedu capsules (particles) are recommended during the SARS-CoV-2 medical observation period, while Qingfeipaidu soup (including Maxingshigan soup, Sheganmahuang soup, Xiaochaihu soup, Wuling powder), Xiyanping injection, Xuebijing injection, Reduning injection, Tanreqing injection, Xingnaojing injection, Shenfu injection, Shengmai injection and Shenmai injection are recommended in the clinical phase [99].

##### SARS-CoV

The Ministry of Science and Technology of China has announced eight Chinese medicines that have been clinically confirmed to improve symptoms in SARS patients: Qingkailing injection, Houttuynia cordata injection, Radix isatidis granules, Xinxue granules, Jinlian Qingre granules, Dengzhanxixin injection, compound Kuh-seng injection and Xiangdan injection. In addition, Qingqi Liangying oral liquid and Qingwen oral liquid, and Jiedu pills, as well as anti-SARS I and anti-SARS II showed effective inhibitory effects on SARS-CoV [157] (Table 3).

**Table 3:** Chinese medicine with active ingredients against HCoV

Type	Chinese medicine	Active ingredients	Mechanism	Reference number
SARS-CoV	*Glycyrrhiza radix*	Glycyrrhizin and derivatives from it	Inhibit the invasion and replication	[128, 129]
	*Lycoris radiata*	Lycorine	N/A	[132]
	N/A	ZZ-1	N/A	[131]
	*Houttuynia cordata*	Polysaccharides and ethyl acetate extracts, such as afzerin and quercetin	Act on the body's immune system with anti-complement activity, and inhibits 3CLpro and RdRp activity	[133, 134, 136]
	*Rheum rhabarbarum*	Emodin	Suppress the interaction between S protein and ACE2 and inhibits 3clpro activity	[135]
	*Polygonum multiflorum*	N/A	Suppress the interaction between S protein and ACE2	[136]
	*Tripterygium regelii*	Quinone-methide triterpenes	Inhibit the enzymatic activity of 3CLpro	[137]
	*Ecklonia cava*	Dieckol	Inhibit the enzymatic activity of 3CLpro	[138]
	*Alnus japonica*	Diarylheptanoids	Inhibit the enzymatic activity of 3CLpro	[139]
	Seeds of *Psoralea corylifolia*	Phenolic phytochemical	Inhibit the enzymatic activity of 3CLpro	[140]
	*Angelica keiskei*	Chalcone 6	Inhibit the enzymatic activity of both 3CLpro and PLpro	[141]
	*Salvia miltiorrhiza*	Tanshinones	Inhibit the enzymatic activity of both 3CLpro and PLpro	[142]
MERS-CoV	Fruits and twigs of *Aglaia foveolate*	Silvestrol	Inhibit viral mRNA cap-dependent translation	[153]
	N/A	Flavonoids such as herbacetin, isobavachalcone, quercetin-3-beta-*O*-d-glucoside and helichrysetin	Inhibit the enzymatic activity of 3CLpro	[154]
SARS-CoV-2	*Folium mori, Atractylodes lancea, Fritillaria, Zingiber officinale, Lonicerae japonicae* flos, Forsythia *suspensa,* and *Amomum tsao-ko*	N/A	Block the binding between ACE2 and viral S protein	[143]
	*Astragalus, Panax ginseng, Dioscorea* spp.*,* and arecae semen	N/A	Regulate RAAS	[144]
	N/A	Quercetin and its derivatives	Block the binding between ACE2 and viral S protein, IL-6R and IL-6	[147]
	*Licorice*, *Ephedra*, *Bupleurum* root	N/A	Block the binding between IL-6R and IL-6	[145, 146]
	*Bupleurum*	Saikoside A and saikoside D	Bind to 3CLpro and block the binding between ACE2 and viral S protein	[148]
	*Scutellaria baicalensis*	Baicalein	Bind to 3CLpro and block the binding between ACE2 and viral S protein	[149]
	N/A	Liquiritin apioside, iridin, liquiritin, forsythiaside, procyanidin B-5, 3′ -o-gallate and saikosaponin C	Inhibit RdRp	[150]
	*Aster, Ligusticum chuanxiong, Salvia miltiorrhiza*	Aster pentapeptide A, ligustrazine, salvianolic acid B	Inhibit the enzymatic activity of 3CLpro	[151]
	Ginger, Ginkgo, Chuanxiong	Gingerol, ginnol, ferulic acid	Inhibit the enzymatic activity of PLpro	[151]
	N/A	Hypericin and baicalein	Bind to nsp14	[152]
HCoV-229E	Leaves of *Euphorbia neriifolia*	3β-Friedelanol	N/A	[155]
	Fruits and twigs of *Aglaia foveolate*	Silvestrol	Inhibit viral mRNA cap-dependent translation	[153]
HCoV-NL63	*Sambucus FormosanaNakai*	Ethanol extract-related caffeic acid	Interfere with the binding of HCoV-NL63 to the receptor of ACE2 and co-receptors such as heparin sulfate proteoglycan	[156]

SARS-CoV: Severe acute respiratory syndrome coronavirus; MERS-CoV: Middle East respiratory syndrome coronavirus; SARS-CoV-2: Severe acute respiratory syndrome coronavirus 2; N/A: not applicable; 3CLpro: 3C-like protease; PLpro: Papain-like protease; RdRp: RNA-dependent RNA polymerase; ACE2: Angiotensin converting enzyme 2; RAAS: Renin–angiotensin–aldosterone system; IL: interleukin; Nsp: non-structural protein; mRNA: messenger RNA

## Discussion

As of November 13, 2020, SARS-CoV-2 had infected 53 218 786 people worldwide and killed a total of 1 301 631 people. Unfortunately, the epidemic is still not under control in many countries. Despite a lack of HCoV-specific drugs, many potent targets for drug discovery have been explored, and many vigorously designed clinical trials are being carried out in an orderly manner. In the present study, we analysed the pathogenesis of and drug therapy targeting seven HCoVs, including four common types (HCoV-229E, -OC43, -NL63, -HKU1) and three highly pathogenic types (SARS-CoV, MERS-CoV, SARS-CoV-2); special attention was given to SARS-CoV-2.

Among the highly pathogenic CoVs, SARS-CoV transmission has been rare since 2004, so clinical trials of drugs and vaccines are difficult to carry out. To date, there are no specific drugs or vaccines against MERS-CoV. mABs, such as m336 [9], lopinavir/ritonavir [30], IFN [98, 99], etc., are potential antiviral drugs against MERS-CoV, but additional evidence is needed to determine their efficacy.

Because COVID-19 is a new, acute, severe infectious disease, the anti-SARS-CoV-2 drug development strategies are to screen existing drugs to identify potentially effective drugs, to expand indications and to develop a vaccine. The safety of conventional drugs has been mostly verified; if effective, they can be quickly applied in clinical practice. To date, thousands of clinical trials of SARS-CoV-2 have been registered worldwide. Hot topics include antiviral drugs such as RaRp inhibitors [51–54, 59], Mpro inhibitors [48, 49], chloroquine and its derivatives [33, 34], viral envelope inhibitors, arbidol [27, 29], and immunotherapy drugs such as IFNs [100] and cytokine storm inhibitors [122, 123, 125–127]. Usually, the duration from initial experimental research to clinical trial completion is long. However, due to the COVID-19 pandemic, many drugs have been entered into clinical trials that are not randomized, controlled, or double-blinded. Their efficacy, toxicity, and side effects are discovered during application. For example, it was previously reported that hydroxychloroquine and chloroquine acted against coronavirus, and the synergistic use of hydroxychloroquine and azithromycin reduced the viral load and improved clinical results. However, later studies found that the heart-related side effects of these drugs included extension of the QT interval, so the WHO terminated the studies [33, 34]. Clinical trials found that lopinavir/ritonavir had a poor effect on COVID-19, while others, such as arbidol [27, 29], remdesivir [51–54], favipiravir [59], IFN-α2b [100], convalescent plasma [119, 120], corticosteroids [122, 123] and tocilizumab [125–127], had different and even opposite results, which can be further validated by experimental evaluation and clinical experience. When the production of inflammatory factors is increased, convalescent plasma, corticosteroids, and tocilizumab should be used early and in appropriate amounts. Because most traditional Chinese medicines are compounds and few single drugs or single active ingredients are used, it is difficult to determine which ingredients are effective in clinical trials. It is hard to differentiate the compounds associated with the mechanism. In a laboratory study of a single active ingredient, glycyrrhizin had a strong inhibitory effect on SARS-CoV-2 [128, 129], which is of great significance for further clinical study. The future research direction for traditional Chinese medicine is to identify and modify a single potent drug or active ingredient and adjust the compound dose and administration method.

This review summarized the conventional and potential drugs at according to each action site, which can improve clinicians’ understanding of the results of current clinical studies to guide clinical decisions. It also enables researchers to understand drug action sites to discover potential effective drugs.

## Conclusions

This review summarized the progress in drugs that inhibit the HCoV proliferation, affect the action of the host's immune system as well as plant-derived Chinese medicines, which not only provides researchers a more comprehensive understanding of the current status of drug development against HCoVs, but also provides directions for further exploration. However, the pharmacological effects and adverse reactions of some drugs under study are still unclear, and hence well-designed high-quality studies are needed to further study the effectiveness and safety of these potential drugs in order to accelerate drug development targeting SARS-CoV-2 and thus promote progress towards ending the pandemic.

## Data Availability

Not applicable.

## Abbreviations

CoV: Coronavirus
HCoVs: Human coronaviruses
SARS: Severe acute respiratory syndrome
MERS: Middle East respiratory syndrome
SARS-CoV-2: Severe acute respiratory syndrome coronavirus 2
SARS-CoV: Severe acute respiratory syndrome coronavirus
MERS-CoV: Middle East respiratory syndrome coronavirus
COVID-19: Coronavirus disease 2019
ARDS: Acute respiratory distress syndrome
IFNs: Interferons
gRNA: Genomic RNA
NSPs: Non-structural proteins
PLpro: Papain-like protease
Mpro: 3C-like protease
RdRp: RNA-dependent RNA polymerase
sgRNA: Subgenomic RNA
ERGIC: ER-Golgi intermediate compartment
RBD: Receptor binding domain
mABs: Monoclonal antibodies
NAAE: *N*-(2-Aminoethyl)-1-aziridineethanamine
DPP4: Dipeptidyl peptidase 4
GRFT: Griffithsin
TMPRSS2: Transmembrane protease serine 2
HR1: Heptad repeat 1 domain
HR2: Heptad repeat 2 domain
NHR: N-terminal heptad repeat
6MP: 6-Mercaptopurine
6TG: 6-Thioguanine
POMs: Polyoxometalates
PTB: Pyrithiobac
WHO: World Health Organization
TRS: Transcriptional regulatory sequence
mRNA: Messenger RNA
NTP: Nucleoside triphosphate
dsRNA: Double-stranded RNA
ADK: Aryl diketoacids
SAM: S-adenosyl-l-methionine
ATA: Aurintricarboxylic acid
siRNA: Small interfering RNAs
DMV: Double-membrane vesicles
DRACO: DsRNA-activated caspase oligomerizer
TGEV: Transmissible gastroenteritis virus
ACE2: Angiotensin converting enzyme 2
RAAS: Renin–angiotensin–aldosterone system
